# Adapting federated cyberinfrastructure for shared data collection facilities in structural biology

**DOI:** 10.1107/S0909049512009776

**Published:** 2012-04-06

**Authors:** Ian Stokes-Rees, Ian Levesque, Frank V. Murphy, Wei Yang, Ashley Deacon, Piotr Sliz

**Affiliations:** a Department of Biological Chemistry and Molecular Pharmacology, Harvard Medical School, Boston, MA 02115, USA; b Howard Hughes Medical Institute, Harvard Medical School, Boston, MA 02115, USA; c Northeast Collaborative Access Team (NE-CAT), Advanced Photon Source, Argonne National Laboratory, Argonne, IL 60439, USA; d Joint Center for Structural Genomics, Stanford Synchrotron Radiation Light Source, Stanford University, Menlo Park, CA 94025, USA

**Keywords:** grid computing, data handling and security, diffraction images, collaborative tools, structural biology

## Abstract

Early stage experimental data in structural biology is generally unmaintained and inaccessible to the public. It is increasingly believed that this data, which forms the basis for each macromolecular structure discovered by this field, must be archived and, in due course, published. Furthermore, the widespread use of shared scientific facilities such as synchrotron beamlines complicates the issue of data storage, access and movement, as does the increase of remote users. This work describes a prototype system that adapts existing federated cyberinfra­structure technology and techniques to significantly improve the operational environment for users and administrators of synchrotron data collection facilities used in structural biology. This is achieved through software from the *Virtual Data Toolkit* and *Globus*, bringing together federated users and facilities from the Stanford Synchrotron Radiation Lightsource, the Advanced Photon Source, the Open Science Grid, the SBGrid Consortium and Harvard Medical School. The performance and experience with the prototype provide a model for data management at shared scientific facilities.

## Abstract 2

It has been difficult, historically, to manage and maintain early-stage experimental data collected by structural biologists in synchrotron facilities. This work describes a prototype system that adapts existing federated cyberinfrastructure technology and techniques to manage collected data at synchrotrons and to facilitate the efficient and secure transfer of data to the owner’s home institution.

## 1. Introduction

The field of structural biology provides atomic-scale models of macromolecules. While these models are typically made public through the Protein Data Bank (PDB; Berman, 2000 ▶), the source experimental data used to establish the models is generally not published. It has been difficult, historically, to manage and maintain this early-stage experimental data, and impractical to make it publicly available. Recent advances in data management systems provide the opportunity to reconsider data retention and publication policies. The shift towards data collection from shared scientific facilities, such as synchrotron beamlines where users from numerous institutions are hosted, compounds the importance of establishing improved storage and data management systems. Remote users, who ship their samples to a facility for data collection, also require more robust data management and movement infrastructure. Advances in the technology and automation at these shared facilities are producing higher data rates, with an anticipated need to process terabytes per day in the near future (Soltis *et al.*, 2008 ▶). These challenges are similar to those faced by genomics research or high-energy physics: centralized data collection at a shared facility by a large group of users with independent affiliations and collaborations. By adapting technology and techniques developed for federated cyberinfrastructure it is possible to significantly improve the operational environment for facility users and administrators. This paper describes a prototype system developed and deployed for the structural biology community that leverages federated identity management systems and grid computing infrastructure to streamline authentication and authorization, data access and data management for multi-gigabyte data sets. These capabilities provide a foundation for secure archival systems that enable collaboration and, optionally, public access to valuable data products of the research process that otherwise are often lost with time or backed up to inaccessible archives.

This prototype focuses on macromolecular crystallography, a sub-discipline of structural biology that has undergone a rapid expansion in recent years. Crystallographic data are routinely collected on specialized X-ray beamlines at shared synchrotron facilities. Each beamline operates 24 h per day and continually produces large sets of images that need to be securely stored and subsequently transferred to the home institution of the research group that provided the sample under investigation.

Here we present a prototype system designed to support members of 170 structural biology laboratories participating in the SBGrid Consortium (http://www.sbgrid.org/). The system integrates with data collection facilities at synchrotron beamlines at the Advanced Photon Source (APS), the Stanford Synchrotron Radiation Lightsource (SSRL) and a shared X-ray data collection facility at Harvard Medical School (HMS). The system relies on Globus Online (Foster, 2011 ▶) to link disparate storage systems, X.509 user certificates (Housley *et al.*, 2002 ▶) for identity tokens, the Virtual Organization Management System (VOMS) (Alfieri *et al.*, 2005 ▶) to define roles and group membership, a MyProxy server (Basney *et al.*, 2005 ▶) as a centralized user credential store (similar to a network-accessible digital key chain), and the SBGrid Science Portal (http://portal.sbgrid.org/) for user account management. As a prototype it demonstrates the viability of deploying a system to address federated data access and archiving of data in a secure manner for researchers, laboratories and collaborations who produce data at shared scientific facilities.

## 2. Materials and methods

The novel work of this prototype is based on the integration of services with the SBGrid Science Portal in order to link user identities, group affiliations, data access and data movement. Conceptually, the portal acts as a central location for users to register and manage group membership. Federated and community driven collaborations are known as virtual organizations (VOs), which can include a hierarchy of sub-VOs and specific roles which group members assume. From the web portal, users are able to access data held on various storage systems: facility-based, institutional, laboratory or personal computer. They can then initiate data movement requests between any two endpoints (for example, from a facility to a laboratory), and the transfer will be scheduled and managed by third-party services that do not require the user to maintain a connection between the two endpoints. Storage systems subscribe to the portal’s group membership lists to manage access control, and configure a mapping from a ‘portal’ identity to the local storage system’s user identifier.

The prototype is based on software and infrastructure provided by the Virtual Data Toolkit (Roy *et al.*, 2009 ▶), the Open Science Grid (Pordes *et al.*, 2007 ▶) and Globus (Foster, 2005 ▶). User identities are handled through the US Department of Energy’s Energy Science Network (ESNet) Certificate Authority (CA) (Muruganantham *et al.*, 2005 ▶) which issues X.509 certificates to eligible users. Group membership is handled by the VO-hosted and managed VOMS server (Alfieri *et al.*, 2005 ▶).

The use of web interfaces to facilitate interaction with federated cyberinfrastructure has been proposed in the form of abstract models (Von Laszewski & Foster, 1999 ▶), generic frameworks (Novotny *et al.*, 2004 ▶; McLennan & Kennell, 2010 ▶) or domain-specific portals (Klimeck *et al.*, 2008 ▶). The development of the Globus Online service (GO) (Foster, 2011 ▶) has now also made the previously complicated task of using high-performance parallel file transfer tools such as GridFTP (Allcock *et al.*, 2001 ▶), often the only means to access storage systems and major computing centers, accessible through simple web interfaces. The existing SBGrid Science Portal, designed specifically for the structural biology community, acts as a central web-based interface for users and groups to manage accounts and data. Group management services are derived from VOMS, with web-based data access control from GridSite (McNab & Li, 2010 ▶), and system-level identity management and access control using the FreeIPA LDAP server.

The X.509 public key infrastructure, common to most grid environments, has been used to provide secure federated identity tokens. A federated computing environment requires a simple and secure mechanism to authenticate users and make authorization decisions. Users need a common portable identification scheme that allows them to access systems at independent computing centers: their home institution; that of their collaborators; and at any facility where they may be collecting, storing or processing data. System administrators need secure mechanisms to delegate authentication, data access control (distinct from system access control) and the organization of users into groups or collaborations. The SBGrid VOMS server provides the authoritative list of group members which can then be used by various GUMS (Baker *et al.*, 2003 ▶) servers (for example, at facilities) or in a grid-mapfile (a text-based configuration file) to assign group members to specific system-level accounts. Individual groups use VOMS to self-administer which users are members of their group, and their roles within the group, providing greater operational autonomy to user groups. In this way users at a shared facility authenticate using their personal grid identity token, rather than a system-level username. This alleviates the need for shared accounts and passwords, while avoiding the need for the facility to create accounts for every user, instead allowing multiple X.509 identity tokens to map to the same shared system-level account. The MyProxy server at the National Center for Supercomputing Applications (NCSA) has been used as an intermediary to cache proxy certificates for users and to make them accessible using the more conventional username/passphrase approach. With MyProxy, users do not need to manage their digital certificates directly, as the process for this is cumbersome and specific to each web browser and operating system.

The SBGrid Science Portal hosts the single identity for all users of the prototype system. This eliminates the need for users to interact with the ESNet Certificate Authority, a VOMS server or MyProxy. Globus Online does not have account interaction application program interfaces (APIs), so a separate GO account must be created and linked to the portal identity. The portal also acts as a storage host, holding user data if necessary. The portal identities are handled by an LDAP-based system, FreeIPA. This single identity source is used for authentication to the science portal, for command-line shell access, and for the shared X-ray data collection facility.

## 3. Results

The trial of the prototype system consisted of configuring SSRL and Northeast Collaborative Access Team (NE-CAT) as GO endpoints, setting up the necessary X.509 authentication system, and mapping grid identities to user identities at the participating sites. SSRL, as part of the Stanford Linear Accelerator Center which already participates in Open Science Grid, already had much of the necessary grid infrastructure in place, including GridFTP-enabled storage systems. NE-CAT, with no previous exposure to federated cyber­infrastructure, utilized the Globus Connect (GC) client and made necessary firewall changes. Users requested grid accounts through the SBGrid Science Portal, which automatically registered them into the SBGrid VO, and created a proxy certificate with the NCSA MyProxy server. The users also had to create Globus Online accounts and link these to their grid certificates.

The system allows users to access and utilize the synchrotron facility in the normal way, saving data to group-specific storage areas local to the beamline. Once collected, users can elect to initiate a data transfer back to their home institution, or leave the data on the facility’s storage system and access it ‘on-demand’ for later analysis and processing. These transfers are mediated by the GO service which acts as a third-party controller. This provides numerous advantages over conventional file transfer mechanisms such as rsync, scp or ftp. The use of GridFTP as the underlying file transfer protocol enables high-performance parallel data transfers to maximize throughput. GO provides a simple web-based interface to initiate third-party file transfers between two sites, monitored by GO, but with the data traffic routed directly between the endpoints. This does not require the user to maintain an active connection to either of the endpoints, nor does it place the user or GO as an intermediary in the network path. GO also implements reliable file transfer which copes with any transient network or connection failures. The use of federated identity tokens allows GO to act on behalf of the user automatically and autonomously to reconnect and retry transfers if they are interrupted using the delegated proxy identity token for the user.

The GC client further aides in this process as it establishes a secure tunneled connection from the client system (a user laptop or desktop, or a laboratory file server) to GO which can then be used to rendezvous the endpoint connection from another GridFTP server or GO ‘private’ endpoint. This is invaluable in situations where firewalls or administrative complexity make it impractical to set up a permanent public GridFTP server at a desired endpoint. A multi-user GC agent is available that runs a single instance of a tunneling GridFTP server but can be configured to support multiple user connections from the web-based GO controller interface. This is designed for persisted operation on file servers that cannot easily be configured with a public GridFTP server.

A representative data set of 2000 X-ray image files, requiring 34 GB of storage, were transferred between three classes of endpoint: beamlines (SSRL and NE-CAT), laboratories (HMS) and data archival facilities [National Center for Supercomputing Applications and Fermi National Accelerator Laboratory (FNAL)]. Table 1 ▶ shows the transfer rates between various pairs of endpoints. In all cases these rates outperformed the alternatives of scp or rsync by 50% to 300%. Completion times ranged from 12 to 25 min with Globus Online, in contrast to 30 to 60 min for conventional transfer techniques.

**Figure 1:**
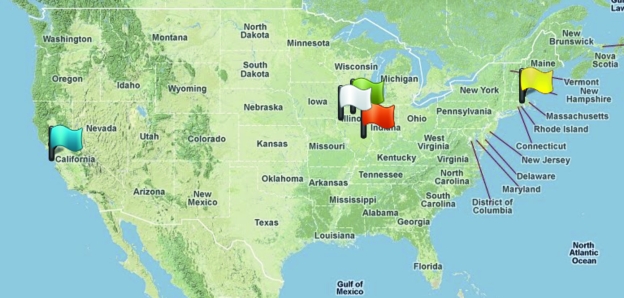
Geographic distribution of five endpoints participating in the trial of the prototype system. The flags represent, from left to right, Stanford Synchrotron Radiation Lightsource (blue, Stanford Linear Accelerator Center, Palo Alto, CA, USA), Fermi National Accelerator Laboratory (white, Batavia, IL, USA), Northeast Collaborative Access Team (green, Advanced Photon Source, Argonne, IL, USA), National Center for Supercomputing Applications (red, University of Illinois Urbana-Champaign, Champaign, IL, USA) and Harvard Medical School (yellow, Boston, MA, USA).

Fig. 1 ▶ illustrates the geographic location of the various endpoints. Although not illustrated in this table, additional trial transfers were made to Texas Tech, the University of Nebraska-Lincoln, the University of California San Diego and Sao Paulo State University (Brazil).

## 4. Discussion

The primary objectives of this project were to prototype improved models for data management at shared X-ray beamlines and to facilitate the efficient and secure transfer of collected data to the owner’s home institution. Secondarily, this project demonstrates aspects necessary for a permanent facility-based data archive, where users and laboratories can reliably access their data ‘on-demand’, without the pressure to store and preserve that data at their home institution.

Tertiary objectives relate to the evaluation of grid computing tools and protocols for managing users, groups, access control and data in a collaborative federated environment. These issues were central to the original development of grid computing over a decade ago (Foster, 2001 ▶).

While systems exist to improve the high-level management of experimental data and the data’s life-cycle (Moore *et al.*, 2006 ▶; Mattmann *et al.*, 2004 ▶; Flannery *et al.*, 2009 ▶), there are limited facilities for simple end-user access and management of federated data sets. The typical solution for beamline users is to transport an external hard drive with them to the facility and to copy files over USB or FireWire. The rapid increase in remote users is eliminating this option. Facilities generally hold user data for a period of weeks to months, but only as a temporary store until the user can repatriate their collected data at which point their institutional or laboratory system administrators take responsibility for managing and backing up the data. In most cases the institutions or laboratories are poorly equipped to be the long-term archivists of these important experimental results, and anecdotal comments suggest the data is discarded within a few years of the departure of the original ‘owner’, or maintained through stacks of disconnected and often unlabelled hard drives. Locating beamline data collected one or two years earlier can often be difficult or impossible. It is to improve this operational reality that the prototype system described here was developed.

Data access control through GO is based on the system-level user that a particular grid identity token is mapped to. On-disk file and directory permissions are applied to any file system operations by this system-level user (POSIX users and file systems). Additional access control mechanisms are available on the SBGrid Science Portal, namely LDAP-based WebDAV, and X.509 DN-based Grid Access Control Language (GACL) (McNab & Li, 2010 ▶). WebDAV has the advantage of being accessible through standard network file system connection protocols; however, managing the access control policies currently requires manual editing of 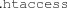 configuration files on a per-directory basis through a command-line interface. In contrast, GACL provides a browser-based ACL editing tool and user-driven DN lists for groups; however, it requires the user to have their certificate available in their browser’s keystore. No other aspect of this system requires users to have direct access to their X.509 certificate on the client side. The single-user GC client is not supposed to require any client-side specific manual system configuration; however, strict firewall rules at NE-CAT prevented it from working without some adjustment: the systems there had no outbound Internet connectivity except for basic services (22, 80, 443 for ssh, http, https, respectively). The multi-user GC client has promising features for supporting laboratory-level storage systems with a tunneled GridFTP server, but was not available in time for this prototype.

Federated cyberinfrastructure requires the organization of users into manageable groups. While the original vision of grid computing was for a VO to represent such a group (Foster, 2001 ▶), the capabilities and requirements of a VO have resulted in relatively static long-lived entities. Smaller user-managed groups, sometimes called dynamic VOs, are possible through several tools. The latest VOMSAdmin interface simplifies the creation of a hierarchy of VOMS groups and the definition of access control lists (ACLs). These ACLs are associated with roles that permit the administration of a single sub-group, or entire sub-trees in the VOMS group hierarchy. Users can then be assigned those roles, and they can self-organize VOMS-group membership. With a GUMS/PRIMA environment, rich mapping policies can be configured based on the user’s X.509 Attribute Certificates (ACs). GUMS, which is installed and used by a single site, can be configured to fetch VOMS information for all recognized VOs from the respective VO’s VOMS servers.

For example, NE-CAT can query the SBGrid VOMS server to obtain a list of VOMS groups (*e.g.* representing laboratory groups or collaborations) within the VO, and a list of those users (by DN) within each VOMS group. NE-CAT then has the option to automate the construction of the grid-mapfile, or to base a mapping decision directly on the VOMS group AC within the user’s X.509 authentication token. A manual protocol is necessary when new groups are added to the VO so NE-CAT can create a new system-level group.

This prototype has motivated several beamlines to consider the benefits of a federated identity and data management environment. The next step is to extend this prototype to a production environment and to include more users, laboratory groups and beamlines. The experience gained in the process has provided some clear guidance around the improved use of VOMS, and a need to evaluate Storage Resource Manager (Shoshani *et al.*, 2002 ▶) software with its fuller feature set as an alternative to the basic GridFTP servers that have currently been deployed. The mechanics of system-level account creation and mapping must also be further improved, likely requiring an evaluation of a GUMS/PRIMA system as an alternative to grid-mapfiles. The lack of ACL visibility and management of files through the GO interface is acceptable in the short term, but enhancement to existing systems or integration of the alternatives mentioned above (WebDAV with LDAP, or GACL) will eventually be required. Expanding the prototype into a long-term archival system would require a greater formalization of archival policies, funding for operational infrastructure and staffing, and consideration of issues such as data set unique identifiers and public release policies. The DataCite initiative (Brase, 2009 ▶), for example, provides a model to issue document object identifiers (DOIs) for data sets that are to be archived and disseminated.

An advantage of using GridFTP and X.509 identity tokens is the ease with which users and data can interact with existing federated cyberinfrastructure for any compute-intensive processing of the data. The authors have shown elsewhere (Stokes-Rees & Sliz, 2010 ▶) the power of this approach, a form of the map/reduce (Dean & Ghemawat, 2004 ▶) paradigm, to parallelize processing, reduce wait time for results, or to enable new, previously intractable, analytical techniques.

Improved data archival systems and public access to the original imaging data would aid the research process, facilitate the development of improved structure determination algorithms by researchers focused on methods, and allow independent validation of the structure. A full featured system would reduce the data management overhead, improve the secure sharing of data, retain valuable early stage data products, and publish a more complete record of the experimental results, ultimately leading to the more efficient production of high-quality models. Publication standards have already evolved to expect the ‘structure factor’ data, which provides a summarized view of the raw experimental data, to be published in addition to the structural model. Unfortunately structure factor data is not sufficient to fully validate the calculation of experimental phases, space group assignment, or the correctness of the data integration strategy. Additionally, data sets that do not lead to publication may still be valuable in the future if they are publicly accessible, possibly leading to new or improved structure models.

Historically, calls to establish experimental data archives for crystallography to hold larger data sets have been met with limited support, mostly owing to the high cost of establishing large community-wide archiving facilities. Such initiatives are now technologically and economically feasible. For example, storing a single-crystal data set for every one of the 67000 deposited macromolecules in the PDB derived from X-ray crystallography would require 130 TB (based on 19 MB per image and 100 images per crystal). Using bulk storage with basic data loss protection this volume of data can be stored for hardware costs of a few tens of thousands of dollars (http://bioteam.net/2011/08/real-world-backblaze-costs/) in less than one server rack. The remaining challenges then focus on security, metadata, annotation, federated user and group management, data provenance and lifecycle, replication and cataloging.

**Figure 2:**
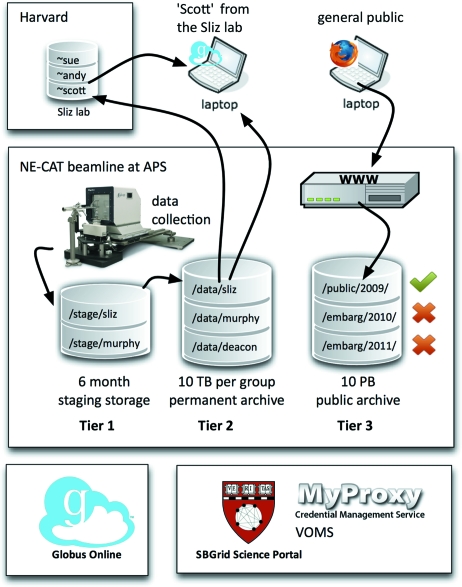
Example usage scenario. User ‘Scott’ from the Sliz Laboratory at Harvard collects data at the NE-CAT beamline. It is stored temporarily to a tier 1 staging server in sliz@necat.aps.argonne.gov:/stage/sliz before being archived to tier 2 storage in sliz@necat.aps.argonne.gov:/data/sliz. Scott can use Globus Online to access his data at NE-CAT and transfer it to his personal space on the laboratory file server scott@sliz.harvard.edu:/home/scott or to his own laptop. Other laboratory members can access his data at NE-CAT owing to the mapping of all Sliz Laboratory members to the same system identity. The general public can access public archived data from 2009 in the tier 3 archival storage through a web interface. More recent archived data from 2010 and 2011 is embargoed and not available to the public.

We envision a three-tier model of time- and quota-limited storage. The first tier would contain full experimental data, be private to the data owners, be retained for less than one year, and be subject to a storage quota. The second tier would be long-term private group storage, also subject to a storage quota, but with a fixed public release date three to five years in the future. The third tier would hold all public data, and be managed by the archive’s curatorial staff rather than the group owner. Pressure on per-group storage quota may encourage data owners to release data as public to move it from tier 2 private storage to tier 3 public storage earlier than the contracted release date. Designated data managers within groups could move data at will from one tier to the next, but not back again. Aspects of this model parallel the process used for structure deposition with the PDB. Fig. 2 ▶ illustrates an example deployment of this prototype, including the NE-CAT facility, it’s shared staging and archival storage system, the laboratory file servers at Harvard, a personal computer, and a user who controls data movement between the systems with the assistance of Globus Online.

To expand the adoption of this prototype system it would be necessary to advance the integration of the SBGrid Science Portal, MyProxy server and Globus Online for integrated account management and secure data access. It would also be necessary to formalize and automate a procedure for shared facilities to approve users or groups from the central VOMS server and to integrate this with their local account creation and X.509 mapping protocols. Achieving this would provide a base for federated data access and high-performance reliable file transfer. Subsequent to this, the issues around group management, annotation, metadata, access control, archival policy, file catalogs and replication/backup would need to be considered. Among these, data security, access control and public deposition protocols would be of key importance. Tertiary issues around information lifecycle management, data set global unique identifiers, provenance, data format details and data collection conditions could also be addressed. Importantly, this archival system is not specific to macromolecular crystallography and could be applied to the many areas where long-lived, high-value and large scientific data sets are present.

The trial of this prototype has established the viability of integrating existing systems, software and models for federated grid computing to improve the operational conditions for shared scientific data collection facilities. Additional capabilities not present in the prototype are variously provided by systems such as iRODS (Moore *et al.*, 2006 ▶), OODT (Mattmann *et al.*, 2004 ▶) and ICAT (Flannery *et al.*, 2009 ▶). Both OODT and ICAT would need to be evaluated for their modularity and capacity to be integrated with the existing SBGrid Science Portal. Issues of long-term support for the facility-based storage systems will need to be addressed, but the proposed combination of a federated environment with centralized archival storage would provide a more sustainable and economical model for the data management needs of this community.

## Figures and Tables

**Table 1:** Transfer rates between participating sites

	Transfer rate (MB s^−1^)				
	To HMS	To SSRL	To NE-CAT	To NCSA	To FNAL
From HMS		47	29	53	45
From SSRL	23		N/A	26	22
From NE-CAT	29	N/A		N/A	N/A
From NCSA	19	20	N/A		N/A
From FNAL	36	39	N/A	N/A	
